# A Modified Design External Fixator System for Calcaneal Fractures: Surgical Technique and an Observational Single-Center Study

**DOI:** 10.3390/jcm15082991

**Published:** 2026-04-15

**Authors:** Michela Saracco, Clara De Negri, Roberta Pagano, Maria Rizzo, Massimo Mariconda

**Affiliations:** 1 Department of Public Health, Division of Orthopedic and Traumatology, University of Naples “Federico II”, 80131 Naples, Italy; clara.denegri@unina.it (C.D.N.); roberta.pagano2@unina.it (R.P.); maria.rizzo@unina.it (M.R.); maricond@unina.it (M.M.); 2 CEINGE Advanced Biotechnology, 80145 Naples, Italy

**Keywords:** calcaneal fracture, heel fracture, external fixation, minimally invasive surgery, surgical technique

## Abstract

**Background:** Displaced intra-articular calcaneal fractures are difficult to treat due to their complex anatomy and the high risk of soft-tissue complications. Although open reduction and internal fixation (ORIF) enables accurate anatomical reconstruction, it can be associated with substantial wound morbidity. Minimally invasive techniques have been developed to mitigate these risks. This study aims to describe a modified external fixation technique and report its clinical and radiographic outcomes in consecutive cases of patients with displaced intra-articular calcaneal fractures, with a minimum follow-up period of one year. **Methods:** The surgical technique is described in detail. The technique was evaluated by analyzing the treatment results in a case series of 17 patients. The time from injury to surgery, radiographic parameters (Böhler’s angle and time to union) and clinical outcomes were recorded and analyzed, as were complication rates. The minimum follow-up period was 12 months. **Results:** Surgical reconstruction was performed at a mean of three days (range 1–6 days; SD 1.5) after injury. Preoperative radiographic parameters showed significant deformity, with calcaneal morphology restored at follow-up. At 12 months, the mean Böhler angle had increased to 27.8°, and all fractures had achieved radiographic union. Functional outcomes improved progressively over time, with increases in both the AOFAS and SF-12 P scores. One complication was observed: K-wire displacement requiring conversion to ORIF in a psychiatric patient. **Conclusions:** The proposed technique facilitates early surgical treatment and the satisfactory restoration of calcaneal morphology with no soft-tissue complications. It appears to be safe and effective in selected patients. A longer follow-up will provide further insight into long-term outcomes, such as subtalar osteoarthritis.

## 1. Introduction

Calcaneal fractures are relatively uncommon, accounting for just 1–2% of all fractures. They are the most common type of fracture affecting the tarsal bones, accounting for almost 60% of cases, and are usually caused by high-energy trauma, such as falling from a great height. These injuries are associated with significant morbidity and long-term functional impairment [1]. Calcaneal fractures are often accompanied by extensive soft-tissue injury, substantially increasing the risk of postoperative complications. The most common fracture pattern is intra-articular fractures involving the posterior facet of the subtalar joint, which are associated with poorer outcomes and a higher incidence of complications, even when adequately treated. Additional risk factors such as osteoporosis, diabetes, peripheral neuropathy, osteomalacia and immunosuppressive therapies further complicate management in elderly patients [2]. The widespread use of computed tomography (CT scanning) has improved understanding of fracture morphology and enabled the development of prognostic classification systems, such as the Sanders classification [3]. Nevertheless, considerable debate persists regarding the indications for surgical treatment and the optimal surgical approach, particularly when comparing open versus minimally invasive techniques. Accurate reduction in fracture fragments and restoration of calcaneal anatomy are crucial to minimizing the risk of malunion and post-traumatic subtalar osteoarthritis. Over the past three decades, open reduction and internal fixation (ORIF) using an extended lateral approach and anatomical plate fixation has been considered the gold standard for many displaced intra-articular calcaneal fractures. However, this approach is associated with a high incidence of complications, including wound dehiscence, infection, hematoma, subtalar stiffness, and injury to the sural nerve or peroneal tendons [4]. To reduce soft-tissue morbidity, several minimally invasive techniques have been developed [5,6]. The method we propose is easy to perform from a technical perspective, does not require specific instruments and, most importantly, prevents varus displacement of the posterior calcaneal process, unlike other methods that have already been described. Furthermore, using 2.5 mm threaded K-wires and an external bar prevents the fixation devices from loosening. The aim of the present study is to describe an external fixation technique with a modified design, with or without the option of a minimally invasive surgical approach to the subtalar joint. The surgical, clinical, and radiographic outcomes of this technique will be evaluated in a consecutive, single-center, case series of patients with displaced intra-articular calcaneal fractures, with a minimum follow-up period of one year. 

## 2. Materials and Methods

### 2.1. Indication for the Proposed Treatment

The proposed modified external fixation technique is indicated for:

- Displaced intra-articular calcaneal fractures (Sanders II–IV) [7] with joint depression, treated in association with percutaneous or minimally invasive reduction techniques, or with loss of hindfoot length, height, or alignment.
- Fractures in which soft-tissue conditions contraindicate an extended lateral approach (e.g., severe edema, blistering, compromised soft-tissue envelope, etc.);
- Polytrauma patients requiring early fixation or damage-control;
- Open fractures requiring temporary or definitive fixation;
- Patients with comorbidities associated with increased wound complication risk (e.g., diabetes, smoking, peripheral vascular disease, etc.);
- Elderly patients with osteoporotic bone, in whom percutaneous fixation with threaded wires, with or without bone graft or substitute, provides adequate stability.

Contra-indications include Sanders IV fractures with severe comminution requiring early subtalar joint arthrodesis.

### 2.2. Pre-Operative Planning

A comprehensive preoperative evaluation includes assessment of soft-tissue conditions and neurovascular status. Standard radiographs (lateral, axial, and Broden views) and CT scans are obtained to evaluate fracture morphology, thalamic depression, comminution, and planning of wire trajectories.

Perioperative management includes:

- Prophylactic antibiotics (cefazolin 2 g intravenously at induction and appropriate additional coverage in case of open fracture);
- Perioperative thromboembolic prophylaxis with low-molecular-weight heparin starting upon hospital admission, stopped 12 h before the procedure and resumed 6 h later.

### 2.3. Surgical Technique

#### 2.3.1. Patient Positioning

**Figure 1:**
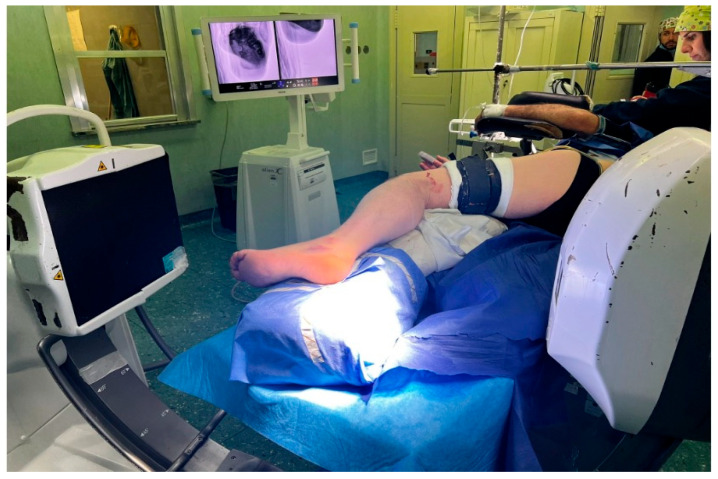
Patient positioning: lateral position.

**Figure 2:**
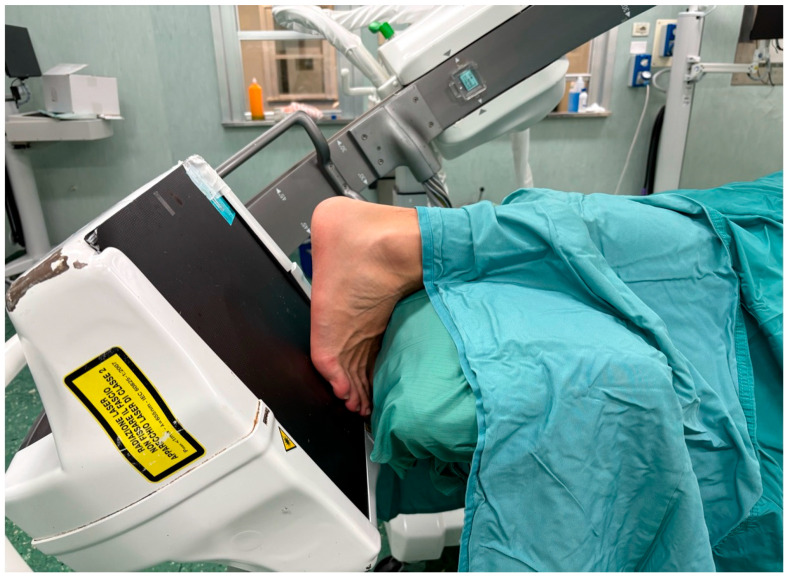
Patient positioning: prone position.

**Figure 3:**
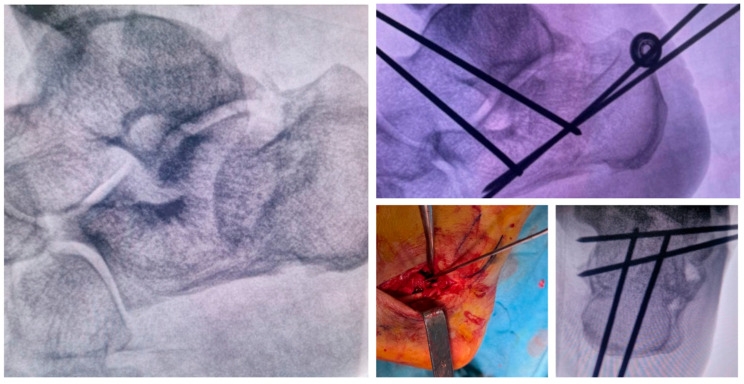
Complex calcaneal fracture treated in the lateral position through the mini-invasive sinus tarsi approach to reduce the subtalar joint. Stabilization was performed percutaneously as described in the surgical technique.

The patient is positioned in either lateral (Figure 1) or prone decubitus on a radiolucent operating table. The prone position facilitates plantar foot positioning under fluoroscopic guidance and allows easier wire insertion (Figure 2), whereas the lateral position is preferred when a minimally invasive open reduction in the subtalar joint is required, such as through a sinus tarsi approach (Figure 3).

#### 2.3.2. Approach

Stabilization is performed percutaneously and may be combined with minimally invasive open reduction techniques. Closed reduction is achieved using K-wires as joysticks. Depressed thalamic fragments may be elevated through a small lateral incision or via a sinus tarsi approach under fluoroscopic control. Bone substitutes can be injected through the same surgical access to provide subthalamic support. Particular care is taken to avoid injury to the sural nerve and peroneal tendons.

#### 2.3.3. Fixation

**Figure 4:**
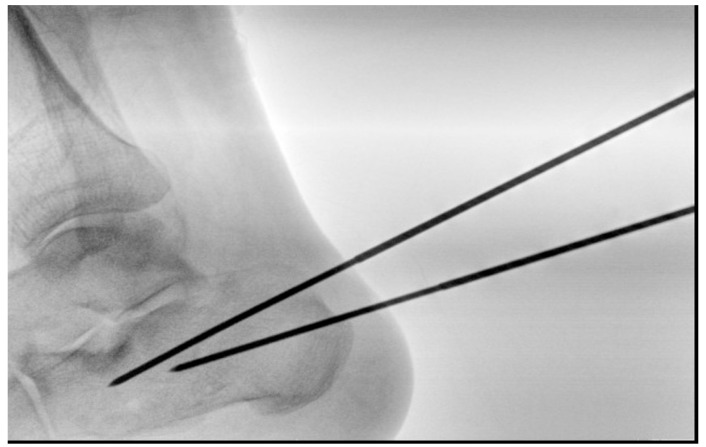
Surgical technique: the first 2 K-wires are inserted from the posterior tuberosity to the anterior process.

**Figure 5:**
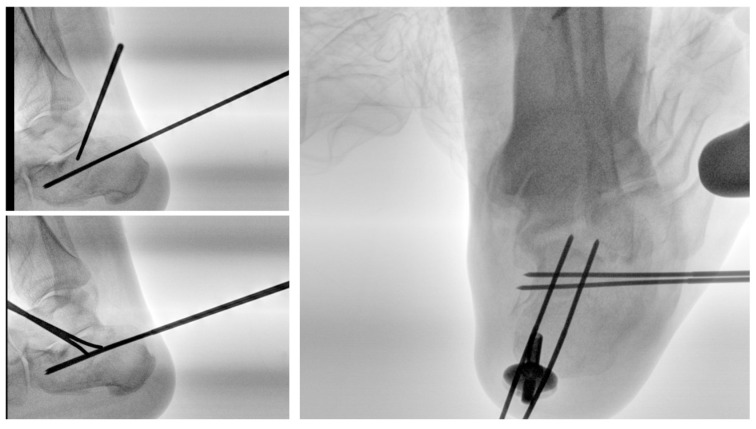
Surgical technique: 2 K-wires are inserted from the lateral side to the medial one, providing support to the subtalar joint.

**Figure 6:**
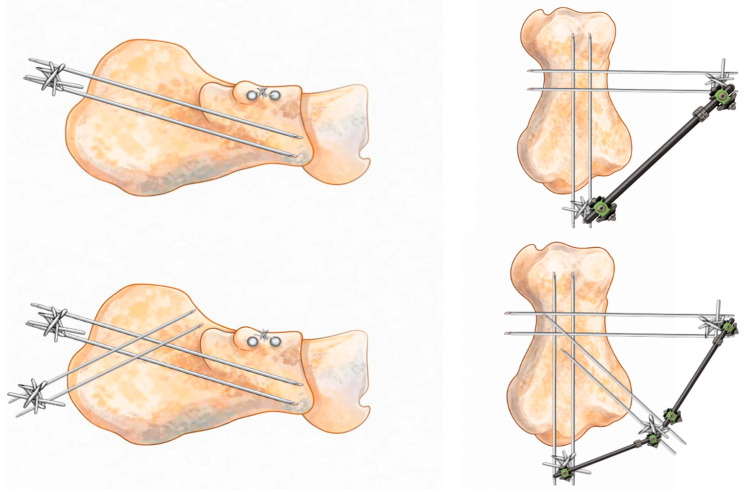
Surgical technique: the proposed ex-fix technique can be set up in 2 different ways, with or without 2 optional K-wires to give further support to the posterior facet in case of greater bone defect/comminution.

**Figure 7:**
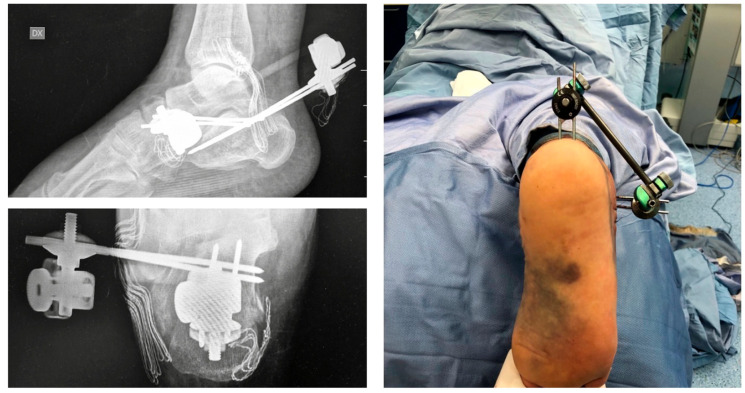
Surgical technique: K-wires are connected externally using clamps and a connecting bar.

Fixation is achieved using four threaded 2.5 mm Kirschner wires:

1. ***Posterior–Anterior Wires (2 wires):***
  Parallel 2.5 mm threaded K-wires are inserted from the posterior tuberosity toward the anterior process or, in cases of anterior process comminution, to the cuboid. These wires maintain calcaneal length and alignment while controlling varus/valgus displacement (Figure 4).

2. **Lateral–Medial Wires (2 wires):**
  Parallel wires are inserted perpendicular to the first ones in the subthalamic region, providing direct support to the elevated thalamic surface and augmenting construct stability (Figure 5).

3. Additional infero-superior wires may be used to further support the posterior facet when required (Figure 6).

4. All wires are connected externally using clamps and a connecting bar, forming a stable mini-external fixator. Threaded wires reduce the risk of migration and loosening (Figure 7).

### 2.4. Biomechanical Rationale

The combination of posterior–anterior and lateral–medial threaded K-wires provides a three-dimensional stabilization of the calcaneus. This construct maintains:

- Hindfoot length, height, and width;
- Posterior facet alignment and subtalar congruity;
- Subthalamic support in the presence of metaphyseal voids.

The external bar and clamps distribute load evenly, while threaded wires prevent pin migration, enabling early controlled motion without compromising stability.

### 2.5. Post-Operative Management

Weekly clinical examinations are performed to monitor wound healing and to prevent superficial infection. Rehabilitation begins on postoperative day 1:

- Active and passive ankle and foot mobilization;
- Isometric strengthening of intrinsic and extrinsic musculature.

The external fixator is removed at approximately 40 days postoperatively in an outpatient setting. Partial weight-bearing is maintained until radiographic bone consolidation. Full weight-bearing is permitted on average 8 weeks post-op, based on fracture healing and patient tolerance.

**Figure 8:**
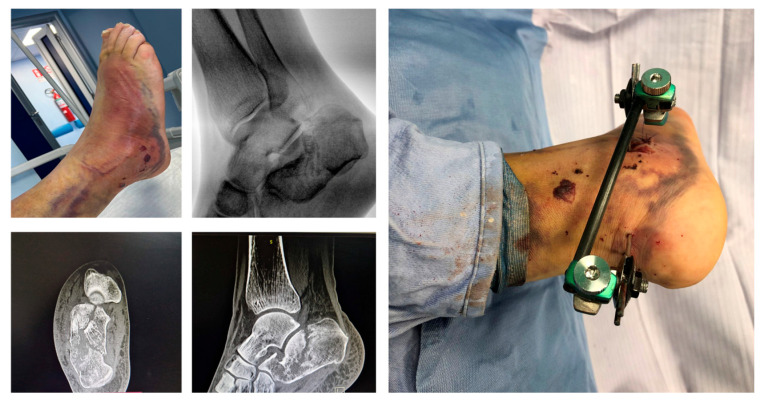
65-year-old male patient affected by displaced calcaneal fracture with soft tissue impairment treated with the proposed ex-fix method.

In Figure 8, we report a 65-year-old male patient affected by a displaced calcaneal fracture treated with the proposed method.

**Figure 9:**
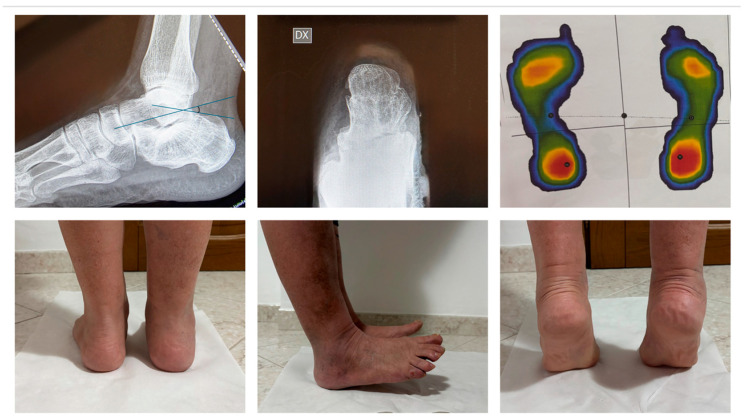
65-year-old male patient at 18 months of F.U.: good clinical and radiographical results. Böhler’s angle was 32.1°. The baropodometric evaluation showed overlapping pressure areas between the two feet.

In Figure 9, the same patient at 18 months of follow-up shows good functional and radiographic results.

## 3. Single-Center Case Series

### 3.1. Methods

We studied a total of 17 (4 women) patients, who underwent surgery for heel fractures with the described technique at a mean follow-up of 13.5 months (12–18 months; SD 3.35).

All the patients included in this study signed an informed consent to participate. All procedures performed were in accordance with the 1964 Helsinki Declaration and its subsequent amendments.

The inclusion criteria were age > 18 years and unilateral closed calcaneal fractures. The exclusion criteria were bilateral procedures, open fractures, revision, pathologic fractures, patients suffering from inflammatory diseases (rheumatoid arthritis) and/or neurological diseases (ictus, chronic diseases such as multiple sclerosis, amyotrophic lateral sclerosis, etc., which affect the ability to full weight-bearing step pattern).

The enrolled patients underwent surgery between March 2023 and September 2024.

During the same period, a total of 36 calcaneal fractures were treated in our department, including two open fractures (1 motorcycle accident and 1 gunshot injury). 17 underwent the described technique. Two patients underwent arthrodesis. Five patients were affected by non-displaced fractures treated conservatively. In 2 cases, the fractures were bilateral. Six patients underwent ORIF. Two patients suffered from rheumatoid arthritis.

The mean age was 56 years (42–65 years; SD 7.71). Three cases were psychiatric patients. Fracture classification included 8 Sanders type 4; 4 Sanders type 3AB; 2 Sanders type 3BC; and 3 Sanders type 3AC. In 10 cases, the right os calcis was involved. A bone substitute to fill the bone gap due to subthalamic articular depression was used in 3 cases.

Patients were followed up regularly at two and four weeks after surgery and then every three months.

Clinical evaluations for this study were carried out on the data collected at 6 months post-surgery, then at the one-year post-operative follow-up visit.

### 3.2. Demographics and Clinical Scales

Patient demographic details were collected and analyzed as well as time between injury and surgery, duration of the surgical procedure and post-operative complications: hardware migration, fracture displacement, blisters, skin complications such as wound and/or pin infection, Clinical evaluations were performed using AOFAS ankle and hindfoot scale [8] and 12-Item Short Form Health Survey (SF-12) [9].

The AOFAS ankle and hindfoot scale (Italian Version) is used to assess clinical outcomes; this scale spans from 0 to a maximum of 100 points (no abnormality) and evaluates pain, function and alignment [10]. The outcomes have been categorized as follows: “Excellent” between 70 and 100, “Good” between 50 and 69, “Fair” between 30 and 49, and “Poor” between 0 and 29.

The 12-Item Short Form Health Survey (SF-12) was used to measure the impact of the pathological condition on the quality of life, evaluating the physical and mental status of patients [9].

Clinical assessments were administered by two different authors, blinded to pre-operative values.

### 3.3. Radiological Evaluations

All the patients underwent standard X-Ray evaluations (antero-posterior, lateral and axial views) and CT-scan after the trauma to evaluate the degree of comminution and articular depression. The CT-scan was also essential for the classification of the fracture according to Sanders (7). X-rays were also regularly taken to assess bone healing and to evaluate the Böhler’s angle [11].

The Böhler’s angle is formed by the intersection of a line drawn from the superior apex of the posterior calcaneal tuberosity to the superior portion of the subtalar articular surface and by a line drawn from the subtalar articular surface to the superior limit of the anterior apophysis of the heel. The angle physiologically measures 20–40 degrees. An angle less than 20° is considered pathognomonic of a fracture [12].

At 6 months and 12 months, full weight-bearing X-rays were obtained.

Radiological evaluations were performed by two independent surgeons on the pre-operative X-Rays and on the one-year post-operative ones. In case of disagreement, the first author sought to resolve the disagreements, if possible.

### 3.4. Potential Sources of Bias and Statistical Analysis

Data was collected using an Excel (Microsoft) sheet and statistical analysis was performed using SPSS (IBM SPSS Statistics 30.0).

To minimize the risk of data collection bias, clinical and radiographic data of the enrolled patients were collected from the department’s database. Similarly, a thorough past medical history was collected upon admission, enabling accurate patient selection considering the exclusion criteria. At the time of the first follow-up, confirmation of the information reported in the patient record was requested, thereby confirming enrolment.

Open fractures were excluded as they could influence the results in terms of complications.

No formal power calculation was performed; all consecutive eligible patients between March 2023 and September 2024 were enrolled.

Demographics are reported using descriptive statistics (mean, range, standard deviation). The Shapiro–Wilk test was used to assess the normality of distribution. The t-test was used to compare parametric parameters (ex. clinical scores). Non-parametric variables were analyzed using the Chi-square test. A statistical confidence level of 95% was selected. A *p*-value < 0.05 determined the statistical significance.

## 4. Results

Patients underwent surgery at a mean time of 3 days (1–6 days; SD 1.5) after injury. The average surgical procedure duration was 36.2 min (30–50 min; SD 6.39). All fractures achieved radiographic bone healing at a mean time of 10.8 weeks (8–14 weeks; SD 2.1).

The mean Böhler’s angle at hospital admission was 20.5 ± 1.57° (16–26; SD 3.3), and it improved at the 12-month follow-up to 27.8 ± 1.14° (25–32; SD 2.4). The improvement of the Böhler’s angle was statistically significant (t: −7.37; *p* < 0.05), as desired among the goals of treatment.

The mean AOFAS score at 6-month follow-up was 50.5 ± 8.65 (20–80; SD 18.2) and 87.7 ± 7.99 (50–100; SD 16.8) at 12-month follow-up. Comparing the results at 6 and 12 months, there was a statistically significant improvement in values (t: −5.68; *p* < 0.05) (Table 1).

The mean SF-12 Physical values at 6-month follow-up were 31.8 ± 9.55 (21.4–40; SD 20.1) and 43.8 ± 8.84 (23.7–60.8; SD 18.6) at 12 months. Also in this case, the difference between the 6-month and 12-month evaluations was statistically significant (t: −2.23; *p* < 0.05).

The mean SF-12 Mental values at 6-month follow-up were 46.7 ± 12.26 (22.4–60; SD 25.8) and 35.7 ± 12.03 (27.4–60.4; SD 25.3) at 12-month follow-up. No statistical significance was noted in this case between 6-month and 12-month follow-up (t: 1.53; *p*: 0.07). In any case, the SF-12 M is a clinical scale that assesses the mental well-being of the patient and can be influenced by multiple factors.

We only recorded one major complication (hardware displacement requiring conversion to ORIF) in a psychiatric patient. In this case, the patient was very non-compliant due to the psychiatric comorbidity, and it is likely that the complication occurred because of this rather than a true failure of the procedure. No minor complications were recorded. Interestingly, no pin-site infections occurred. Although the sample size is small, we believe this is due to the use of threaded K-wires, which are more stable. The absence of micro-movement would explain this significant finding. All other patients had resumed their normal daily activities, including sports, within 10 weeks. No patients were lost to follow-up.

## 5. Discussion

Calcaneal fractures are often associated with soft tissue impairment immediately after injury. Blisters, swelling and large bruises in the lateral region of the hindfoot are common. Surgical procedures can worsen the condition of soft tissues. Open reduction in these fractures is associated with a high incidence of postoperative complications, such as wound dehiscence or infection [13].

Sometimes, skin lesions are so severe that hardware removal is required, and an orthoplastic approach is necessary to address soft tissue loss. Fracture fragments can also damage the overlying soft tissues, particularly in tongue-type calcaneus fractures. In these cases, surgical reduction is an emergency procedure to avoid subsequent complications, which are often serious [14].

Additionally, local soft tissue swelling requires time to resolve, which can delay surgery by days or even weeks [15].

This makes calcaneal trauma surgery difficult to manage pre- and post-operatively. However, the possibility of a less invasive approach to this type of fracture offers the prospect of managing even complex fractures with a much lower risk of failure related to postoperative complications, as recently reported by Peng et al. [16].

Calcaneal fractures were once predominantly treated non-surgically, as the risk of complications, especially in the short term, was higher in patients undergoing surgery. However, the long-term functional outcome for these patients remained poor, particularly due to the development of post-traumatic subtalar osteoarthritis [17].

The extended lateral approach is widely regarded as one of the primary techniques for treating calcaneal fractures. It enables fracture fragments to be reduced effectively through direct visualization [18]. However, the incidence of wound necrosis with this approach ranges from 2% to 27% (13). In order to reduce complications related to soft tissue trauma, various minimally invasive techniques for treating calcaneal fractures have been evaluated [19,20].

First described in the 1970s by Hackstock et al. and Decoulx et al., these minimally invasive techniques began to gain ground in the early 2000s [21,22]. Several techniques are described and reported in Table 2 [23,24,25,26,27,28,29,30,31,32,33,34,35,36,37,38,39,40,41].

Numerous studies report good clinical and radiographic results of percutaneous fixation methods, if correctly performed. For example, Abdelgaid was able to achieve good to excellent scores using closed reduction and percutaneous fixation [42]. Thor et al. also report satisfactory results in their recent paper [43].

The authors believe that readers will find it meaningful to compare our results with those of the percutaneous technique that most closely resembles ours—namely, percutaneous fixation with elastic wires (MIROS system), as proposed by Battaglia et al. [30]. They reported the clinical and radiographic outcomes of a cohort of 40 patients. Analyzing the 12-month follow-up data, we obtained similar outcomes: our 12-month Böhler’s angle was 27.8 ± 1.14° versus 24 ± 14°, and our 12-month AOFAS score was 87.7 ± 7.99° versus 88 ± 9°. Similarly, patients in both studies resumed activities within ten weeks of injury. However, Battaglia et al. reported a higher number of complications, including three cases of sinus tarsi syndrome and one case requiring subtalar fusion.

Our study shows that a minimally invasive external fixation technique can achieve favorable functional and radiographic results while reducing the overall rate of complications. The early timing of surgery reflects the technique’s minimal impact on soft tissues, allowing prompt intervention without waiting for edema to resolve. In addition, the studied group achieved good correction of hindfoot morphology.

The study aimed to evaluate the efficacy and reliability of our technique in treating patients with calcaneal fractures associated with subthalamic involvement. Another objective was to emphasize the reduced incidence of infectious complications or soft tissue damage following treatment with a minimally invasive approach.

Although numerous studies have demonstrated the superiority of percutaneous techniques over traditional ones, doubts remain about the ability to ensure anatomical reduction in the fracture and maintain it with the fixation tools used [44,45].

Based on the results obtained by the authors in this case series, the achievement of satisfactory radiographic bone healing seemed evident. The Böhler’s angle following surgery was re-established and >20° in all treated patients. From a clinical perspective, using the evaluation of the AOFAS and SF-12 scales, all the patients, except for one, reported good clinical results with a low risk of complications.

In patients with fractures associated with skin impairment, surgery with extended lateral approaches with internal fixation can lead to skin necrosis and an increased risk of infections and osteomyelitis, especially if performed early, as said before. However, this does not pose a problem in the case of external fixation. In our study, in fact, the average interval between trauma and surgical treatment was three days, significantly reducing the time to return to the patient’s daily activities.

Our results agree with other published case series. Gao et al. evaluated the treatment of calcaneal fractures with percutaneous Kirshner wire fixation by analyzing the Böhler’s angle, VAS scale and Maryland Foot Score in a cohort of 19 patients: the Böhler’s angle was found to be on average 23.7°, with good clinical outcomes [46]. Cui et al. also evaluated the treatment of calcaneal fractures with a percutaneous system in a cohort of 14 patients. The results obtained are comparable to those reported in our study, despite the longer time interval between trauma and surgical procedure (5.4 days) [47]. Other methods, as described by Nosewicz et al., used minimal incisions to achieve satisfactory reduction, and later, in their case, internal fixation using cannulated screws in a cohort of 22 patients. In this case, too, the restoration of the Böhler’s angle (mean 29°) was achieved. What appears evident is the surgical site infection reported in 3/22 patients, supporting even more the possibility of greater complications in case of stabilization with internal fixation [48].

The proposed fixation method has several strengths: the threaded wires reduce the risk of loosening compared to non-threaded ones or short pins. The restored calcaneal length and achieved reduction are effectively maintained with large-diameter (2.5 mm) threaded K-wires, representing a more reliable construct than similar ones using smaller-diameter, unthreaded elastic wires [30], reducing the risk of secondary displacement. The use of a connecting bar and external clamps helps maintain the rigidity of the construct. In addition, our construct guarantees greater stability and a reduced risk of varus displacement of the posterior fragment compared to external fixators that use short pins, especially in comminuted fractures [29]. The proposed method could also be used in association with other techniques, such as calcaneoplasty with a balloon system and bone substitute grafting [38].

Our study also has some limitations: first, the absence of a control group. It was not possible to compare the results obtained in the treatment of calcaneal fractures with thalamic depression using minimally invasive methods with patients treated with traditional open approaches or different percutaneous fixation methods. Another limitation of the study is the small size of our sample.

It will be important in the future to add more patients to our preliminary study to increase the statistical significance of the obtained results. Furthermore, the time to follow-up is short and therefore long-term complications such as post-traumatic osteoarthritis remain unknown. Therefore, it would be interesting to follow operated patients over time. Case–control studies will also be useful in demonstrating the non-inferior efficacy of our system compared to others.

## 6. Conclusions

The proposed modified external fixator technique for displaced intra-articular calcaneal fractures was able to achieve good clinical and radiographic results with no soft-tissue complications. It appears to be safe and effective in selected patients, particularly valuable in those with compromised soft tissues or comorbidities predisposing to wound complications. The findings of our study represent preliminary evidence on a modified technique, and further comparative or prospective studies are needed to establish non-inferiority versus other minimally invasive or open approaches to intra-articular calcaneal fractures. In the same way, long-term outcomes remain unknown and require longer follow-up.

## Figures and Tables

**Table 1:** AOFAS outcomes—“Excellent” 70–100; “Good” 50–69; “Fair” 30–49; “Poor” 0–29.

	6-Months Follow-Up	12-Months Follow-Up
1	Good	Excellent
2	Good	Excellent
3	Fair	Excellent
4	Poor	Good
5	Fair	Excellent
6	Excellent	Excellent
7	Good	Excellent
8	Good	Good
9	Excellent	Excellent
10	Poor	Good
11	Fair	Excellent
12	Good	Excellent
13	Good	Excellent
14	Fair	Good
15	Excellent	Excellent
16	Fair	Good
17	Excellent	Excellent

**Table 2:** Percutaneous calcaneal fixation techniques described in the literature.

Authors	Year	Described Percutaneous Technique
Talarico et al. [23]	2004	External ring fixation
Pezzoni et al. [24]	2009	Temporary arthrodesis with K-wires: the “Brixian Bridge” technique
Marsh et al. [25]	2011	Combined screws and K-wires
Mauffrey et al. [26]	2012	Inflatable bone tamp and K-wires
Takahashi et al. [27]	2013	Ilizarov external fixator
Xia et al. [28]	2014	Percutaneous plate via Sinus Tarsi approach
Corina et al. [29]	2014	Mini-calcaneal external fixator with short pins
Battaglia et al. [30]	2015	Percutaneous fixation with elastic wires (MIROS system)
El-Desouky et al. [31]	2017	Super-cutaneous locked plate fixation
Vicenti et al. [32]	2018	Calcaneoplasty (bone tamp filled with tricalcium phosphate) and temporary K-wires (removed 7 days p.o.)
Grun et al. [33]	2020	Arthroscopically assisted percutaneous osteosynthesis
Ebrahimpour et al. [34]	2021	Percutaneous screw fixation
Dai et al. [35]	2021	Cannulated screw fixation and calcium sulphate cement grafting
Dai et al. [36]	2022	K-wires fixation via Sinus Tarsi approach
Yuan et al. [37]	2023	Robot-assisted screw fixation
Delmon et al. [38]	2023	Balloon calcaneoplasty
Schippers et al. [39]	2024	Intramedullary nailing
Basal et al. [40]	2025	Delta frame triplanar external fixator
Liao et al. [41]	2025	Tailored distractor-assisted percutaneous fixation

## Data Availability

The original contributions presented in this study are included in the article/Supplementary Material. Further inquiries can be directed to the corresponding author.

## Supplementary Materials

The following supporting information can be downloaded at: https://www.mdpi.com/article/10.3390/jcm15082991/s1, STROBE Statement—Checklist of items that should be included in reports of cohort studies.
